# Adenosine Encapsulation and Characterization through Layer-by-Layer Assembly of Hydroxypropyl-*β*-Cyclodextrin and Whey Protein Isolate as Wall Materials

**DOI:** 10.3390/molecules29092046

**Published:** 2024-04-29

**Authors:** Yudie Jin, Suning Zhang

**Affiliations:** School of Perfume and Aroma Technology, Shanghai Institute of Technology, Shanghai 201418, China; yudie_j@163.com

**Keywords:** adenosine, hydroxypropyl-β-cyclodextrin, whey protein isolate, layer-by-layer assembly, stability

## Abstract

Adenosine, as a water-soluble active substance, has various pharmacological effects. This study proposes a layer-by-layer assembly method of composite wall materials, using hydroxypropyl-β-cyclodextrin as the inner wall and whey protein isolate as the outer wall, to encapsulate adenosine within the core material, aiming to enhance adenosine microcapsules’ stability through intermolecular interactions. By combining isothermal titration calorimetry with molecular modeling analysis, it was determined that the core material and the inner wall and the inner wall and the outer wall interact through intermolecular forces. Adenosine and hydroxypropyl-β-cyclodextrin form an optimal 1:1 complex through hydrophobic interactions, while hydroxypropyl-β-cyclodextrin and whey protein isolate interact through hydrogen bonds. The embedding rate of AD/Hp-β-CD/WPI microcapsules was 36.80%, and the 24 h retention rate under the release behavior test was 76.09%. The method of preparing adenosine microcapsules using composite wall materials is environmentally friendly and shows broad application prospects in storage and delivery systems with sustained release properties.

## 1. Introduction

Adenosine (AD) ($C_{10}H_{13}N_{5}O_{4}$) is a nucleoside that involves the binding of adenine and ribose. It exists in various forms within biological cells and is produced by the metabolism of adenosine triphosphate (ATP) and participates in ATP synthesis within mitochondria. It plays a crucial role in the energy production and utilization system in the human body [1,2]. AD has a wide range of pharmacological effects, including antiwrinkle, antioxidant, anti-inflammatory, and antitumor effects [3,4]. Lipid formulations have been widely employed in various dermal and transdermal drug delivery systems since the 1970s. Combining adenosine with lipids can improve its permeability in the skin [4,5,6]. Nanotechnology has emerged as an effective approach to improve the physicochemical and biological properties of drugs, including enhancing solubility, providing controlled release, reducing toxicity, and enhancing the specificity of the therapeutic mechanism [7]. AD’s extremely short half-life in the body hinders its application as a central nervous system treatment [8]. To solve this problem, scientists have used various methods to control the release of adenosine [9]. Alice Gaudin et al. [10] have found that the bioconjugation reaction between adenosine and squalene becomes pharmacologically effective and can form nanoparticles with a size of about 120 nm. This component can prolong the adenosine cycle and activate adenosine receptors (ARs), providing neuroprotection in mouse stroke and rat spinal cord injury models. Sooho Yeo et al. [4] used free fatty acids to produce AD-loaded lipid nanoparticles (LNs). By investigating the permeability of AD, they found that LNs significantly increased the permeability of AD. LNs penetrated 16.13% within 8 h, which is approximately 5 times that of the AD solution. Therefore, to prolong the release of adenosine, one can reduce the average daily release rate and extend the release duration. Marie Rouquette et al. [11] proposed a method to coat solid adenosine reservoirs with silk fibroin to maximize drug loading and control the release rate by adjusting the silk fibroin coating. This approach achieved nearly constant adenosine release within 14 days, providing an innovative system for adenosine delivery. However, the pre-treatment process of silk fibroin protein is cumbersome, and the use of organic reagents such as methanol increased experimental safety concerns. While these innovative delivery approaches have potential clinical applications [12], their safety is also of great importance. For example, in the early preparation process of nanoparticles, researchers used chemical methods for encapsulation, and the participation of organic reagents (tert-butyldimethylchlorosilane TBDMSCL, dimethylformamide DMF) did not meet natural and safe conditions [10]. Additionally, some studies have employed chemical and physical enhancers (iontophoresis and sonophoresis) to improve the skin permeability of poorly permeable drugs, which will lead to higher costs of specialized equipment and a series of safety issues related to skin barrier function [4]. When designing drug delivery systems, it is critical to improve the efficacy of adenosine drug delivery and reduce its potential negative effects.

Hydroxypropyl-β-cyclodextrin (Hp-β-CD) is a hydroxypropyl derivative of natural β-cyclodextrin, with a molecular structure composed of seven d-glucopyranose units. It exhibits excellent safety and non-toxicity in animal models, while having lower hemolytic effects [13]. Compared to hydrophobic β-cyclodextrin, Hp-β-CD possesses higher water solubility and better solubilizing and encapsulating properties due to its attached hydroxypropyl groups [14,15,16,17,18]. Therefore, Hp-β-CD has broad and promising application potential in the pharmaceutical and food industries [19,20,21,22,23,24,25]. Hp-β-CD can also be used for AD encapsulation to improve the solubility, bioavailability, and stability of the AD drug [26]. By incorporating 2-Hp-β-CD-adenosine microcapsules synthesized by physical mixing and microwave methods into a hydrogel network [2], the issue of burst release can be addressed, and the release behavior of conventional drugs can be optimized. Cyclodextrins can also be used to form composite coatings with natural high-molecular-weight substances such as chitosan [9] and whey protein for encapsulation purposes. Whey protein isolate (WPI) is mainly composed of β-lactoglobulin (β-LG), α-lactalbumin (α-LA), and bovine serum albumin (BSA), and it exhibits excellent film-forming properties [27], making it suitable as a microcapsule wall-forming material. It is commonly used for encapsulating volatile essential oils, sunscreens, etc. [28,29], and it can interact with various small molecules or biomacromolecules, showing ligand binding and metal ion binding capabilities [30]. Swetank Y Hundre et al. [31] used WPI and β-CD as wall materials to prepare vanillin microcapsules using a spray freeze-drying technique (SFD). The results showed that WPI-containing SFD microcapsules had a spherical appearance with numerous micropores on the surface and exhibited good rehydration properties. However, the particle size of these microcapsules is too large, making it more challenging to prevent leakage in cosmetic applications.

In order to improve the stability of encapsulation and achieve sustained-release capability in terms of skin penetration under specific conditions, this study used Hp-β-CD and WPI as composite wall materials to layer-by-layer [17] encapsulate adenosine. Cyclodextrin microcapsules are often widely used to improve the absorption capacity of drugs. However, the use of intermolecular forces to achieve layer-by-layer assembly of adenosine with cyclodextrin derivatives and whey protein isolate and research on the formation mechanism and interaction of multiple structural microcapsules are still very limited [31,32]. This study focuses on the formation mechanism and interactions of the microcapsules and investigates the release characteristics based on intermolecular forces. The results show that using natural composite materials as wall materials to assemble the drug AD layer by layer through intermolecular forces can increase stability and improve the sustained-release capability of AD, which provides a new path for designing drug delivery.

## 2. Results and Discussion

### 2.1. Molecular Modeling

**Figure 1:**
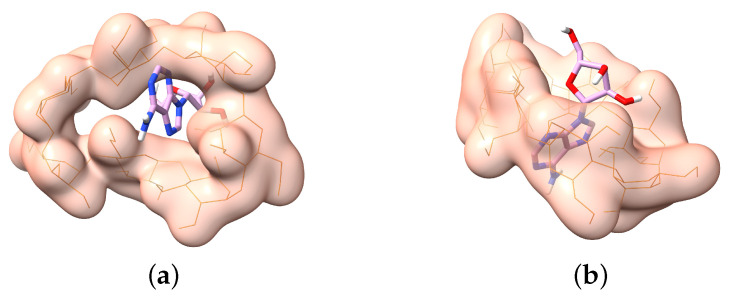
A 3D view of the docking of the AD rod model (C atoms in purple, H atoms in white, O atoms in red, N atoms in blue) with Hp-β-CD (orange lines) monomer, generated using ChimeraX. (**a**) A top view of the docking between AD and Hp-β-CD. (**b**) A side view of the docking between AD and Hp-β-CD.

Molecular docking is a computational technique designed to effectively predict non-covalent binding between large molecules (receptors) and small molecules (ligands) and is an insightful method for studying intermolecular interactions. This technique simulates molecular interactions based on molecular structure, incorporating factors such as hydrogen bonding, van der Waals forces, and charge transfer, to assess the affinity and interaction patterns between receptors and ligands. Molecular docking has been applied in drug design, protein function studies, enzyme catalytic mechanisms, and other fields, providing crucial tools and a theoretical basis for drug discovery and the design of bioactive molecules [33]. We used this method to explore the binding mechanism between AD and Hp-β-CD. AD is a nucleoside compound composed of adenine and ribose, and, structurally, its non-polar moieties primarily include the adenine ring and the carbon chain connecting the ring structures. The binding between AD and Hp-β-CD originates from the hydrophobic interaction between the non-polar moieties of AD and the hydrophobic cavities within the Hp-β-CD molecule. The flexibility of the AD molecule enhances its ability to properly position the non-polar moieties into the cavities, thus facilitating a tight binding by hydrophobic interaction. Figure 1 shows a schematic diagram of the side view of the mutual binding between AD and Hp-β-CD, indicating that AD and Hp-β-CD form a 1:1 complex.

**Figure 2:**
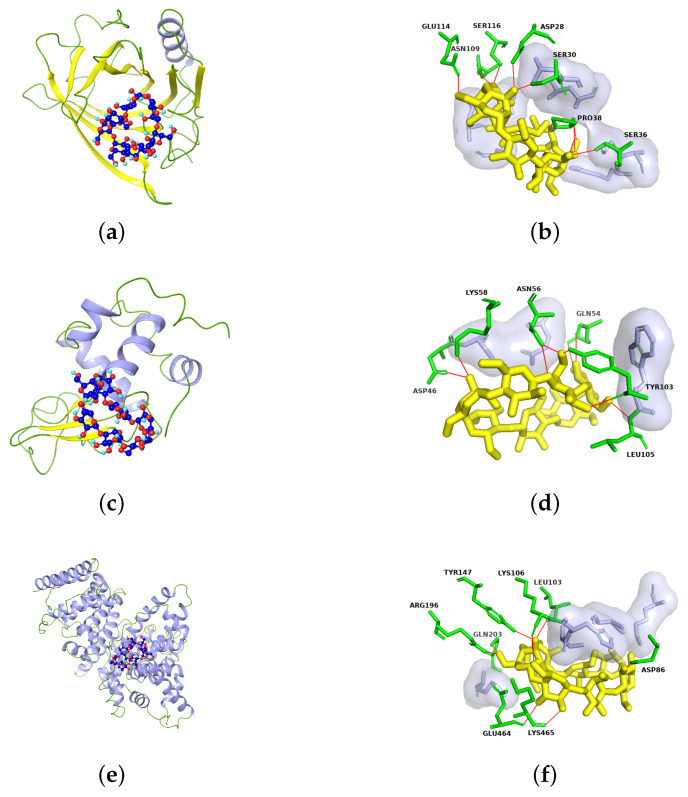
The docking results of Hp-β-CD with (**a**) β-LG, (**c**) α-LA, and (**e**) BSA as visualized by Maestro. The rod-and-ball models represent Hp-β-CD (C atoms in dark blue, O atoms in red, H atoms in sky blue). The specific amino acid residues involved in the interaction with Hp-β-CD (yellow) were identified using Pymol for (**b**) β-LG, (**d**) α-LA, and (**f**) BSA. Key amino acid residues are shown in green, hydrophobic interaction residues are shown in purple, and hydrogen bond interactions are shown as red lines.

Figure 2 depicts the optimal binding positions, interacting amino acids, and related forces for the docking of Hp-β-CD with β-LG, α-LA, and BSA. According to the docking results, the key amino acid residues involved in the binding of β-LG, α-LA, and BSA with Hp-β-CD include 14 residues (SER30, SER36, PRO38, ASN109, SER116, GLU114, ASP28, LEU31, LYS60, LYS69, ASN90, ALA86, ASN88, LEU39), 9 residues (ASP46, LYS58, LEU105, GLN54, ASN56, TYR103, SER47, GLU49, TRP104), and 13 residues (ARG196, GLN203, LEU103, TYR147, ASP86, LYS465, GLU464, LYS106, LYS204, SER104, LYS93, CYS101, HIS105), respectively. Among these interactions, hydrogen bonds are the most prevalent. Specifically, SER30, SER36, PRO38, ASN109, SER116, GLU114, ASP28 in β-LG; ASP46, LYS58, LEU105, GLN54, ASN56, TYR103 in α-LA; and ARG196, GLN203, LEU103, TYR147, ASP86, LYS465, GLU464, LYS106 in BSA are able to form hydrogen bonds with Hp-β-CD. The non-polar cavities within Hp-β-CD can interact with the hydrophobic residues of proteins [34], while the external hydrophilic portions engage in hydrogen bonding with the amino acid residues on the protein surface. According to the docking results, Hp-β-CD can spontaneously bind with the three soluble proteins in whey, namely, β-LG, α-LA, and BSA.

### 2.2. Isothermal Titration Calorimetry (ITC)

**Figure 3:**
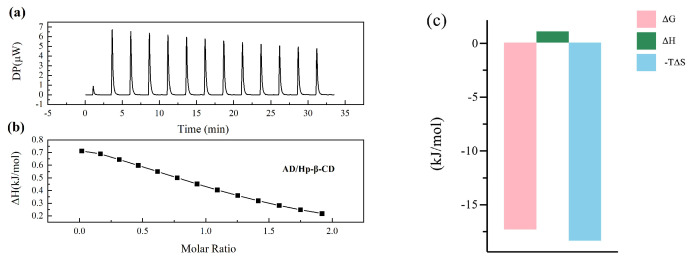
The titration of 1.5 × 10^4^ μM AD with 1.5 × 10^3^ μM Hp-β-CD. (**a**) The heat generated by titrating the same volume of AD into Hp-β-CD solution in a single step. (**b**) The non-linear fitting curve of the binding between AD and Hp-β-CD. (**c**) The thermodynamic changes during the titration of AD with Hp-β-CD.

Isothermal titration calorimetry (ITC) has been widely recognized as the most reliable method for studying molecular interactions in solution and is commonly employed to assess interactions between ligands and large molecules [35]. It is particularly useful for characterizing the interactions between cyclodextrins and guest molecules [36,37,38,39,40,41]. From the ITC of AD with Hp-β-CD shown in Figure 3a, each peak represents the heat generated from a single titration of the same volume of AD into the Hp-β-CD solution. Figure 3b is a non-linear fitting plot of AD binding to Hp-β-CD. It is evident that the reaction heat of the interaction between AD and Hp-β-CD is positive, with all peaks facing upward, indicating the occurrence of an endothermic reaction. Throughout the titration process, as AD gradually occupies the binding sites on Hp-β-CD, the endothermic nature of the peak decreases and eventually reaches saturation. Based on the thermodynamic principles and experimental results outlined by Ross et al. [42], the following conclusions can be drawn from the principles of isothermal titration calorimetry: (1) when ΔH > 0 and ΔS > 0, hydrophobic force primarily governs the interaction; (2) when ΔH < 0 and ΔS < 0, van der Waals forces and hydrogen bonding predominantly drive the interaction; (3) when ΔH < 0 and ΔS > 0, electrostatic force mainly drives the interaction; (4) when the enthalpy change is close to zero or relatively small, hydrogen bonding is typically the driving force. The dissociation constant (KD) signifies the binding affinity between molecules, with KD > 10 μM indicating low binding affinity, 0.01 μM < KD < 10 μM indicating moderate binding affinity, and KD < 0.01 μM indicating high binding affinity [35]. The N value represents the stoichiometry of the binding, while ΔH, ΔS, and ΔG correspond to enthalpy change, entropy change, and Gibbs free energy, respectively. It is evident from the thermodynamic parameters shown in Table 1 that the KD is 917 μM, indicating a low binding affinity between AD and Hp-β-CD. The negative ΔG signifies the spontaneous nature of the binding between AD and Hp-β-CD, and the stoichiometry of their interaction is approximately 1:1. As can be seen from Figure 3c, both ΔH and ΔS are positive, and the value of TΔS significantly exceeds the value of ΔH. This indicates that entropy change contributes significantly to the Gibbs free energy variation, emphasizing that the binding process is primarily entropy-driven. Moreover, the substantial impact of hydrophobic forces suggests that the dominant driving force between AD and Hp-β-CD is hydrophobic interaction.

**Figure 4:**
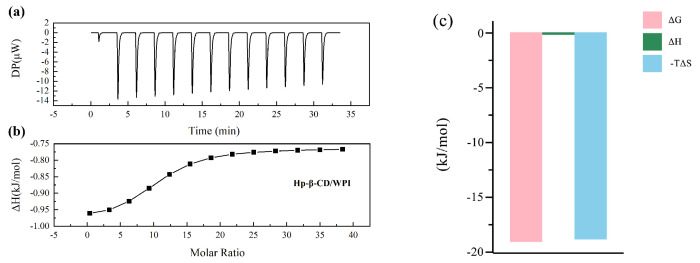
The titration of 6 × 10^4^ μM Hp-β-CD with 3 × 10^2^ μM WPI. (**a**) The heat generated by titrating the same volume of Hp-β-CD into WPI solution in a single step. (**b**) The non-linear fitting curve of the binding between Hp-β-CD and WPI. (**c**) The thermodynamic changes during the titration of Hp-β-CD with WPI.

As shown in Figure 4a, each peak represents the heat generated by a single titration of the same volume of Hp-β-CD into the WPI solution. Figure 4b is a non-linear fitting plot of the binding between Hp-β-CD and WPI solution. It is evident that the reaction heat for the Hp-β-CD and WPI solution interaction is negative, causing the peaks to face downward. This indicates an exothermic reaction, and the reaction ceases when titration reaches saturation over time. From the data in Table 1, the KD value for Hp-β-CD and WPI is 451 μM, indicating low binding affinity. The stoichiometric ratio of Hp-β-CD to WPI is 10:1, and, as shown in Figure 4c, the negative ΔG indicates that the reaction can proceed spontaneously, while the very small and close-to-zero ΔH indicates that the interaction between Hp-β-CD and WPI is primarily driven by hydrogen bonding.

### 2.3. ^1^H NMR Studies

**Figure 5:**
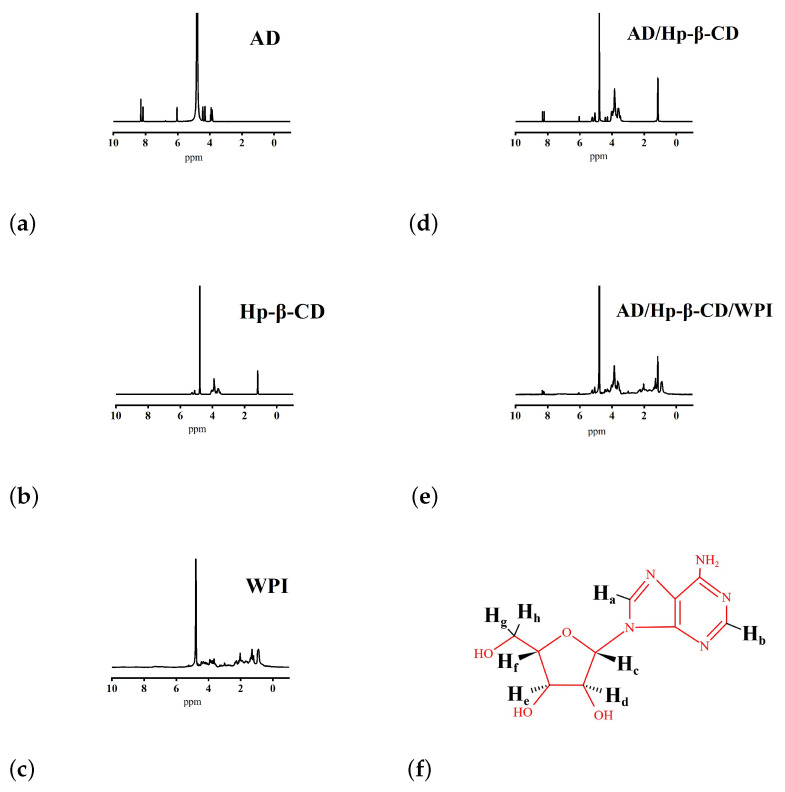
(**a**) AD, (**b**) Hp-β-CD, (**c**) WPI, (**d**) AD/Hp-β-CD microcapsules, and (**e**) AD/Hp-β-CD/WPI microcapsules ^1^H NMR spectra, and (**f**) adenosine chemical structure.

^1^H NMR was used to study the formation of AD/Hp-β-CD/WPI microcapsules, and information on the host–guest interactions between Hp-β-CD, WPI, and the AD drug was obtained through changes in chemical shifts. $D_{2}O$ served as the solvent reference, with a chemical shift of 4.790. Figure 5a–e show the ^1^H NMR spectra of AD, Hp-β-CD, WPI, AD/Hp-β-CD microcapsules, and AD/Hp-β-CD/WPI microcapsules, respectively, while Figure 5f shows the chemical structure of AD. The differences in proton chemical shifts Δδ (δComplex-δfree) for free AD, AD/Hp-β-CD microcapsules, and AD/Hp-β-CD/WPI microcapsules are summarized in Table 2.

Chemical shifts are caused by differences in resonance frequencies due to changes in electron cloud density around different hydrogen nuclei, resulting in shifts in resonance absorption peaks. The positive and negative signs indicate low-field and high-field shifts, respectively. When moving from a high field to a low field, the shielding effect gradually diminishes, leading to an increasing chemical shift. The characteristic peaks of AD at 8.2916, 8.1573, 6.7542, 6.0392, 4.4361, 4.3032, 3.9358, and 3.8495 ppm, respectively, correspond to $H_{a}$, $H_{b}$, $H_{c}$, $H_{d}$, $H_{e}$, $H_{f}$, $H_{g}$, and $H_{h}$ of the adenosine structure (as shown in Figure 5f). It can be seen from Table 2 that the characteristic peaks of AD shifted during the incorporation process. The aromatic protons $H_{a}$ and $H_{b}$ of AD shifted towards the lower field (Δδ = +ve), while $H_{c}$, $H_{d}$, $H_{e}$, $H_{f}$, $H_{g}$, and $H_{h}$ shifted towards the higher field. This is attributed to the partial entry of AD molecules into the Hp-β-CD cavity, which restricted free rotation, while the hydrophobic binding reduced the electron cloud density around AD hydrogen nuclei. From Table 2, it can also be seen that the AD/Hp-β-CD/WPI microcapsules, due to the hydrogen bond effects between Hp-β-CD and WPI, further reduced the electron cloud density around $H_{a}$ and $H_{b}$, the hydrogen nuclei of AD. As the shielding effect decreased, the $H_{a}$ and $H_{b}$ values of AD in AD/Hp-β-CD/WPI showed a more significant shift towards the lower field compared to those of AD in AD/Hp-β-CD. Meanwhile, $H_{e}$ also underwent a slight shift towards the lower field. This is attributed to the fact that, when WPI is enveloped in the outer layer, a small amount of WPI can spontaneously engage in hydrophobic interactions with the portion of AD molecules that did not enter the Hp-β-CD cavity, resulting in a subtle shift in $H_{e}$ [9]. In Figure 5e, the characteristic peaks of WPI, Hp-β-CD, and AD appear in the spectrum of AD/Hp-β-CD/WPI microcapsules. In addition, a noticeable decrease in the AD peak was observed, indicating significant encapsulation, from which it can be inferred that the formation of the ternary complex microcapsules has occurred, revealing the binding morphology of the components.

### 2.4. Fourier Transform Infrared (FTIR) Spectroscopy

**Figure 6:**
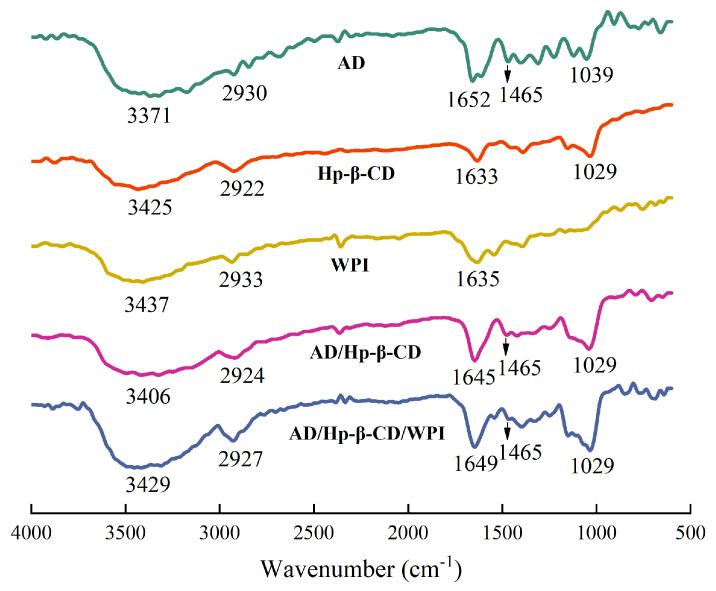
FTIR spectra of AD, Hp-β-CD, WPI, AD/Hp-β-CD microcapsules, and AD/Hp-β-CD/WPI microcapsules.

Figure 6 shows the FTIR spectra of AD, Hp-β-CD, WPI, AD/Hp-β-CD microcapsules, and AD/Hp-β-CD/WPI microcapsules. As shown in Figure 6, the FTIR spectrum of AD exhibits a characteristic peak at 3371 ${\mathrm{cm}}^{-1}$, representing the broad -OH feature; 2930 ${\mathrm{cm}}^{-1}$ is the C-H stretching vibration peak of the adenosine ribose group; 1652 ${\mathrm{cm}}^{-1}$ is the NH bending vibration peak; 1465 ${\mathrm{cm}}^{-1}$ corresponds to the C=N stretching peak of the aromatic ring; and at 1039 ${\mathrm{cm}}^{-1}$, the characteristic peak is associated with the C-O stretching vibration.

The FTIR spectrum of Hp-β-CD, as depicted around 3425 ${\mathrm{cm}}^{-1}$, exhibits a broad characteristic band of -OH. At 1633 ${\mathrm{cm}}^{-1}$, a distinctive peak is observed, corresponding to the deformation band of H-O-H, indicating the attachment of water molecules to Hp-β-CD. In addition, at 1029 ${\mathrm{cm}}^{-1}$, there is a stretching vibration peak related to C-O-C. In the FTIR spectrum of WPI, the absorption peak at 3437 ${\mathrm{cm}}^{-1}$ corresponds to the stretching vibration of -OH. The peak at 2933 ${\mathrm{cm}}^{-1}$ is the stretching vibration of C-H bonds, and at 1635 ${\mathrm{cm}}^{-1}$, the absorption peak is attributed to the C=O stretching vibration of the amide I band. Compared to Hp-β-CD, the -OH peaks of AD/Hp-β-CD and AD/Hp-β-CD/WPI shift from 3425 ${\mathrm{cm}}^{-1}$ to 3406 ${\mathrm{cm}}^{-1}$ and 3429 ${\mathrm{cm}}^{-1}$, respectively. The C-H peak of Hp-β-CD at 2922 ${\mathrm{cm}}^{-1}$ shifts to 2924 ${\mathrm{cm}}^{-1}$ in AD/Hp-β-CD and 2927 ${\mathrm{cm}}^{-1}$ in AD/Hp-β-CD/WPI, indicating the involvement of -OH and alkyl C-H in bonding interactions. Additionally, the NH bending vibration peak of AD shifts from 1652 ${\mathrm{cm}}^{-1}$ to 1645 ${\mathrm{cm}}^{-1}$ in AD/Hp-β-CD and 1649 ${\mathrm{cm}}^{-1}$ in AD/Hp-β-CD/WPI, suggesting its participation in interactions. Furthermore, the C=N stretching peak of the aromatic ring at 1465 ${\mathrm{cm}}^{-1}$ weakens in AD/Hp-β-CD and AD/Hp-β-CD/WPI, indicating the involvement of the aromatic ring in π–π stacking interactions. This phenomenon may be attributed to the encapsulation of AD into the Hp-β-CD cavity, leading to a reduction in its absorption intensity.

### 2.5. Morphology and Size Distribution of Microcapsules

**Figure 7:**
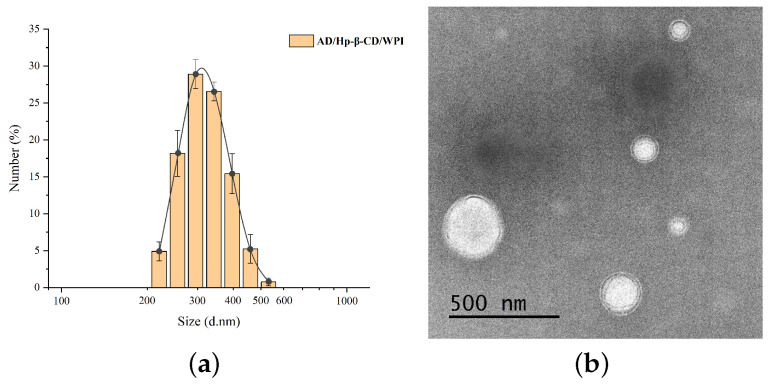
(**a**) Particle size distribution of AD/Hp-β-CD/WPI microcapsules. (**b**) Transmission electron image of AD/Hp-β-CD/WPI microcapsules.

As can be seen from Figure 7a, the particle size distribution of AD/Hp-β-CD/WPI microcapsules is in the range of 200–600 nm, with an average particle size of 311.7 nm and a PDI of 0.151, indicating that the particle distribution is relatively uniform. The TEM image in Figure 7b shows that the microcapsules are regular spherical in shape and have a multi-layer core-shell structure, thus confirming the layered assembly structure of the ternary composite microcapsules.

### 2.6. Embedding Rate of Adenosine

It can be determined by a UV–visible spectrophotometer that the content of AD in 0.12 g of microcapsules is 4.932 mg and the content of unencapsulated AD on the surface of the microcapsules is 3.117 mg. According to Equation (1), the encapsulation efficiency of AD in the AD/Hp-β-CD/WPI prepared by layer-by-layer assembly is approximately 36.80%.

### 2.7. Release Behavior Test of Microcapsules

**Figure 8:**
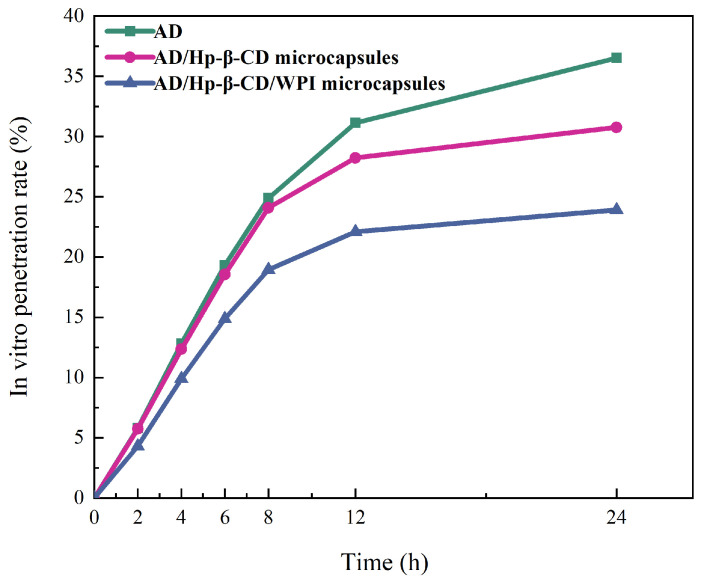
The release behavior of AD, AD/Hp-β-CD microcapsules, and AD/Hp-β-CD/WPI microcapsules.

PBS buffer was used as the receiving solution, and samples were taken regularly over 24 h to measure the UV absorbance at 259 nm to study the release behavior of AD microcapsules. As shown in Figure 8, the permeability of AD, AD/Hp-β-CD microcapsules, and AD/Hp-β-CD/WPI ternary composite microcapsules decreased sequentially over 24 h, and the retention rate of AD/Hp-β-CD/WPI microcapsules reached 76.09%. When AD was dissolved in water, it spontaneously bonded with Hp-β-CD through hydrophobic interactions. Upon the addition of WPI, WPI forms hydrogen bonds with the outer layer of Hp-β-CD in the AD/Hp-β-CD microcapsules. At this time, a synergistic effect was established between hydrophobic interactions and hydrogen bonds [43], which enhanced the stability of the ternary composite microcapsules, thus improving its sustained release performance.

## 3. Experiment

### 3.1. Materials

Adenosine (AD) was purchased from Adamas-beta^®^, abbreviated as Adamas (Shanghai, China). Hydroxypropyl-β-cyclodextrin (Hp-β-CD) was obtained from Meryer^®^ Biochemicals Co., Ltd. (Shanghai, China), and whey protein was purchased from Aladdin^®^ Biochemicals Co., Ltd. (Shanghai, China). Whey protein isolate (WPI) is mainly composed of β-lactoglobulin (β-LG), α-lactalbumin (α-LA), and bovine serum albumin (BSA). Among them, β-LG accounts for approximately 65%, α-LA accounts for about 20%, and BSA accounts for about 6%. All experiments were performed using deionized water. Chemicals were used as received without further purification.

### 3.2. Synthesis of Microcapsules

AD/Hp-β-CD/WPI ternary composite microcapsules were prepared through a layer-by-layer assembly process. Firstly, AD and Hp-β-CD (1:1) were placed in a beaker, 50 mL of deionized water was added, and the mixture was stirred in a 50 °C water bath for 2 h to obtain an AD/Hp-β-CD aqueous solution. This solution was then spray-dried [44] (inlet air temperature of 150 °C, outlet air temperature of 65 °C) to obtain dried AD/Hp-β-CD microcapsules. Subsequently, an appropriate amount of whey protein was added to a beaker with an equal amount of deionized water, stirred in a 30 °C water bath for 12 h, and centrifuged at 3500 rpm for 10 min, and the supernatant was collected. After filtration with a 0.22 μm membrane, it was added to the prepared AD/Hp-β-CD aqueous solution. The mixture was stirred at 50 °C for 30 min and then spray-dried under the same conditions to obtain dried AD/Hp-β-CD/WPI microcapsules. The product was sealed and stored for subsequent characterization.

### 3.3. Characterization of Microcapsules

#### 3.3.1. Molecular Modeling

Through molecular modeling, non-covalent binding between large molecules (receptors) and small molecules (ligands) can be effectively predicted based on molecular structure and intermolecular forces. The 3D crystal structures of Hp-β-CD (PubChem CID: 56972821) and AD (PubChem CID: 60961) were obtained from PubChem https://pubchem.ncbi.nlm.nih.gov/ (accessed on 1 November 2023). The molecular positions of two molecules in Structure Data File (SDF) format were adjusted using Avogadro-1.2.0 software under the Universal Force Field (UFF) mode. Hydrogenation and charge addition were performed using AutoDockTool-1.5.6 software to generate a Protein Data Bank with charges and atom types (PDBQT) files, facilitating the docking of AD and Hp-β-CD molecules. ChimeraX-1.6.1 software was employed for visualizing the results [32] and further refining the docking outcomes. These methods were used to simulate the binding of AD molecules with Hp-β-CD molecules to form microcapsules, and, through modeling, the optimal ratio of the two molecules to bind with each other can be determined. The composition and structure of a whey protein determines its suitability as a carrier. These functions are mainly provided by β-lactoglobulin (β-LG), α-lactalbumin (α-LA), and bovine serum albumin (BSA), so the study chose these three proteins for molecular modeling study [30]. The interactions between Hp-β-CD and the proteins β-lactoglobulin (β-LG), α-lactalbumin (α-LA), and bovine serum albumin (BSA) within whey protein isolate (WPI) were also analyzed through corresponding modeling. The crystal structures of β-LG (PDB: 3NPO), α-LA (PDB: 1F6R), and BSA (PDB: 4F5S) were downloaded from the Research Collaboratory for Structural Bioinformatics Protein Data Bank http://www.rcsb.org (accessed on 24 December 2023) [45]. Protein preprocessing involving procedures such as monomer extraction, the removal of crystalline water, the completion of missing atoms and disulfide bonds, and hydrogenation was performed using AutoDockTool-1.5.6 software PDBQT file operations, including merging non-polar hydrogens, assigning polar hydrogens (including Gasteiger charges), and selecting rotatable bonds [46]. The most likely binding sites of Hp-β-CD on three proteins were explored using Maestro 12.9 software (for academic use only). Pymol-2.1.0 software was used to visualize the results [47] and also as a tool to analyze relevant interacting residues.

#### 3.3.2. Isothermal Titration Calorimetry (ITC)

Isothermal titration calorimetry (ITC) measurements were performed using a PEAQ-ITC microcalorimeter (Malvern, MicroCal Inc., Malvern, UN, USA) at 298.15 K. All solutions were prepared in distilled water and degassed by sonication for 10 min. First, the titration of AD and Hp-β-CD was performed. Solutions of AD and Hp-β-CD at different ratios were prepared, with concentrations expressed in molar concentration [M]. [Syr] represents the AD solution in the syringe, while [Cell] refers to the Hp-β-CD solution in the sample cell. The high-concentration solution in the syringe was directly titrated into the sample cell under continuous stirring [48]. Subsequently, the titration of Hp-β-CD and WPI was carried out, where [Syr] represents the solution of Hp-β-CD inside the titration syringe and [Cell] represents the solution of WPI in the sample cell. The syringe rotation speed was set at 750 rpm. Thirteen titrations were conducted, with each titration lasting 6 s. The initial single titration was 0.4 μL, followed by 12 consecutive titrations of 3 μL each. The titration experiments were conducted in high-feedback mode with a reference power of 4.184 × 10^−5^ J/s. Other experimental parameters included initial delay and injection intervals set at 60 s and 150 s, respectively. Binding isotherms were constructed by plotting the heat generated during each injection, and each measurement was repeated three times. MicroCal PEAQ-ITC Analysis Software-1.41 was used for data analysis and fitting to obtain the binding affinity (in the form of the binding constant KD), interaction mechanism (in terms of binding enthalpy ΔH, binding entropy ΔS, Gibbs free energy ΔG), and stoichiometry of the interaction (N) [49]. ΔH and ΔS were used to determine the type of intermolecular binding, while ΔG was used to determine the spontaneity of the binding.

#### 3.3.3. ^1^H NMR Studies

The ^1^H NMR spectra of AD, Hp-β-CD, AD/Hp-β-CD microcapsules, and AD/Hp-β-CD/WPI ternary microcapsules were acquired using a nuclear magnetic resonance spectrometer (Qone AS400, China). The AD, Hp-β-CD, AD/Hp-β-CD microcapsules, and AD/Hp-β-CD/WPI ternary microcapsules were weighed at 25 mg each and dissolved in 0.55 mL of $D_{2}O$, respectively. All NMR experiments were carried out at 298 K using $D_{2}O$ as the solvent.

#### 3.3.4. Fourier Transform Infrared (FTIR) Spectroscopy

The FTIR spectra of AD, Hp-β-CD, WPI, AD/Hp-β-CD microcapsules, and AD/Hp-β-CD/WPI ternary microcapsules were measured using a Fourier Transform Infrared Spectrometer (Vertex 70, Bruker, Germany). KBr powder was dried in an oven at 140 °C for at least 6 h and then sealed and mixed with the samples for tablet pressing. The spectra were recorded in the wavenumber range of 600–4000 ${\mathrm{cm}}^{-1}$, with a resolution of 4 ${\mathrm{cm}}^{-1}$. Changes in characteristic peaks before and after microcapsule encapsulation were observed through spectral analysis.

#### 3.3.5. Morphology and Size Distribution of Microcapsules

TEM images of the composite were characterized using a transmission electron microscope (Tecnai G2 20 TWIN, FEI Company, Hillsboro, OR, USA). The AD/Hp-β-CD/WPI microcapsule solution was dropped onto a copper grid and dried for TEM measurements. The diameter and size distribution of the microcapsules were measured using a multi-angle particle sizer and a high-sensitivity Zeta potential analyzer (Zetasizer, Nano ZS, Malvern Panalytical Ltd., Malvern, UN, USA).

#### 3.3.6. Determination of Embedding Rate

A total of 0.12 g of AD/Hp-β-CD/WPI microcapsules was taken for high-temperature curing at 135 °C for 15 min, and then the cured microcapsules were placed in a dialysis bag and placed in a beaker with water as the solvent. After 24 h, the liquid was removed from the beaker, and the surface unembedded AD content of the microcapsules was measured at 259 nm using a UV spectrophotometer. The embedding rate of the microcapsules was calculated based on the total AD content in the microcapsule samples and the unembedded AD content on the surface of the microcapsules using the following formula:

(1) $$\mathrm{Embedding}\;\mathrm{Rate}=\frac{\left(W_{1}-W_{2}\right)}{W_{1}}\times 100\%$$

where $W_{1}$ represents the total content of AD in the microcapsules (mg) and $W_{2}$ represents the unembedded AD content on the surface of the microcapsules (mg).

#### 3.3.7. Release Behavior Test of Microcapsules

The release behavior of AD/Hp-β-CD/WPI microcapsules was evaluated using a Franz diffusion cell. A Millipore filter is a semipermeable membrane artificial skin similar to pigskin, which can selectively allow specific substances to pass through and filter particles of specific sizes. The Millipore filter was clamped between the donor and receptor compartments of the Franz diffusion cell, with an effective permeation area of 2.2 ${\mathrm{cm}}^{2}$ and a receptor chamber volume of 6.5 mL. Using PBS buffer as the receptor solution, the Franz diffusion cell was maintained at 37 ± 0.2 °C with a stirring speed of 550 rpm in a water bath. The test samples (with a total concentration of 10 mg/mL, dissolved in deionized water) were added to the donor compartment. Samples (0.2 mL) were withdrawn from the receptor chamber at predetermined time intervals and replaced with an equal volume of fresh PBS buffer. The concentration of adenosine in the receptor chamber was measured at 259 nm using a UV spectrophotometer.

## 4. Conclusions

In this study, AD/Hp-β-CD/WPI microcapsules were prepared using a layer-by-layer assembly method, and the controlled release behavior of the microcapsules was investigated. Molecular modeling and isothermal titration calorimetry results demonstrated the spontaneous molecular interactions between AD and Hp-β-CD, as well as between Hp-β-CD and WPI. A one-dimensional nuclear magnetic resonance hydrogen spectrum and Fourier transform infrared spectroscopy confirmed that AD molecules could partially enter the hydrophobic cavity of Hp-β-CD, followed by further multi-layer composite assembly with WPI. The embedding rate of AD/Hp-β-CD/WPI microcapsules was 36.80%. The release behavior of the encapsulated compounds showed that compared with AD/Hp-β-CD, the layer-by-layer-assembled AD/Hp-β-CD/WPI microcapsules exhibited better stability and could prolong the metabolic cycle of AD and achieve a sustained release effect. The method of preparing AD microcapsules using composite coatings through layer-by-layer assembly is environmentally friendly and biocompatible, and it offers potential for controlled release under specific conditions. This approach holds promise for application in the storage and transportation systems of microcapsules and further research and development in the field of the controlled release of pharmaceutical compounds.

## Figures and Tables

**Table 1:** The thermodynamic parameters of the interactions between AD and Hp-β-CD and Hp-β-CD and WPI.

Parameter	AD/Hp-β-CD	Hp-β-CD/WPI	Unit
N	1.18	10	
KD	917	451	μM
ΔH	1.04	−0.23	kJ/mol
ΔG	−17.36	−19.13	kJ/mol
ΔS	0.06	0.06	kJ/mol/K
−TΔS	−18.40	−18.90	kJ/mol

**Table 2:** ^1^H NMR chemical shift values for free and complexed AD drugs.

AD Protons	AD δFree (ppm)	AD/Hp-β-CD δComplex	AD/Hp-β-CD ΔδComplex-Free	AD/Hp-β- CD/WPI δComplex	AD/Hp-β- CD/WPI ΔδComplex-Free
$H_{a}$	8.2916	8.3184	0.0268	8.3300	0.0384
$H_{b}$	8.1573	8.2125	0.0552	8.2309	0.0736
$H_{c}$	6.7542	6.0446	−0.7096	6.0770	−0.6772
$H_{d}$	6.0392	5.1468	−0.8924	5.1514	−0.8878
$H_{e}$	4.4361	4.4174	−0.0187	4.4420	0.0059
$H_{f}$	4.3032	4.2848	−0.0184	4.2878	−0.0154
$H_{g}$	3.9358	3.8457	−0.0901	3.8635	−0.0723
$H_{h}$	3.8495	3.6087	−0.2408	3.6551	−0.1944

## Data Availability

Data are contained within the article.

## References

[1] Kazemzadeh-Narbat M., Annabi N., Tamayol A., Oklu R., Ghanem A., Khademhosseini A. (2015). Adenosine-associated delivery systems. J. Drug Target..

[2] Vaid V., Jindal R. (2022). RSM-CCD optimized in air synthesis of novel kappa-carrageenan/tamarind kernel powder hybrid polymer network incorporated with inclusion complex of (2-hydroxypropyl)-*β*-cyclodextrin and adenosine for controlled drug delivery. J. Drug Deliv. Sci. Technol..

[3] Xia B., Wang J. (2019). Adenosine inhibits ovarian cancer growth through regulating rhogdi2 protein expression. Drug Des. Dev. Ther..

[4] Yeo S., Kim D., Park M., Woo H.R., Yun J.M., Lee J. (2020). Improved Transport of Adenosine Incorporated in Lipid Nanoparticles across Reconstructed Human Epidermis. Bull. Korean Chem. Soc..

[5] Rouquette M., Lepetre-Mouelhi S., Couvreur P. (2019). Adenosine and lipids: A forced marriage or a love match?. Adv. Drug Deliv. Rev..

[6] Jijie R., Barras A., Boukherroub R., Szunerits S. (2017). Nanomaterials for transdermal drug delivery: Beyond the state of the art of liposomal structures. J. Mater. Chem. B.

[7] Cavalcanti I.D.L., Junior F.H.X., Magalhães N.S.S., Nogueira M.C.d.B.L. (2023). Isothermal titration calorimetry (ITC) as a promising tool in pharmaceutical nanotechnology. Int. J. Pharm..

[8] Gaudin A., Lepetre-Mouelhi S., Mougin J., Parrod M., Pieters G., Garcia-Argote S., Loreau O., Goncalves J., Chacun H., Courbebaisse Y. (2015). Pharmacokinetics, biodistribution and metabolism of squalenoyl adenosine nanoparticles in mice using dual radio-labeling and radio-HPLC analysis. J. Control. Release.

[9] Chen W., Zeng W., Wu Y., Wen C., Li L., Liu G., Shen L., Yang M., Tan J., Zhu C. (2014). The Construction of Tissue-Engineered Blood Vessels Crosslinked with Adenosine-Loaded Chitosan/*β*-Cyclodextrin Nanoparticles using a Layer-by-Layer Assembly Method. Adv. Healthc. Mater..

[10] Gaudin A., Yemisci M., Eroglu H., Lepetre-Mouelhi S., Turkoglu O.F., Dönmez-Demir B., Caban S., Sargon M.F., Garcia-Argote S., Pieters G. (2014). Squalenoyl adenosine nanoparticles provide neuroprotection after stroke and spinal cord injury. Nat. Nanotechnol..

[11] Rouquette M., Lepetre-Mouelhi S., Dufrançais O., Yang X., Mougin J., Pieters G., Garcia-Argote S., IJzerman A.P., Couvreur P. (2019). Squalene-adenosine nanoparticles: Ligands of adenosine receptors or adenosine prodrug?. J. Pharmacol. Exp. Ther..

[12] Pritchard E.M., Szybala C., Boison D., Kaplan D.L. (2010). Silk fibroin encapsulated powder reservoirs for sustained release of adenosine. J. Control. Release.

[13] Davis M.E., Brewster M.E. (2004). Cyclodextrin-based pharmaceutics: Past, present and future. Nat. Rev. Drug Discov..

[14] Truzzi E., Rustichelli C., de Oliveira Junior E.R., Ferraro L., Maretti E., Graziani D., Botti G., Beggiato S., Iannuccelli V., Lima E.M. (2021). Nasal biocompatible powder of Geraniol oil complexed with cyclodextrins for neurodegenerative diseases: Physicochemical characterization and in vivo evidences of nose to brain delivery. J. Control. Release.

[15] Radu C.D., Parteni O., Ochiuz L. (2016). Applications of cyclodextrins in medical textiles. J. Control. Release.

[16] Gao S., Liu Y., Jiang J., Ji Q., Fu Y., Zhao L., Li C., Ye F. (2019). Physicochemical properties and fungicidal activity of inclusion complexes of fungicide chlorothalonil with *β*-cyclodextrin and hydroxypropyl-*β*-cyclodextrin. J. Mol. Liq..

[17] Zhao Y., Wang Y., Zhang Z., Li H. (2023). Advances in controllable release essential oil microcapsules and their promising applications. Molecules.

[18] Chang C., Song M., Ma M., Song J., Cao F., Qin Q. (2023). Preparation, Characterization and Molecular Dynamics Simulation of Rutin–Cyclodextrin Inclusion Complexes. Molecules.

[19] Qiu N., Li X., Liu J. (2017). Application of cyclodextrins in cancer treatment. J. Incl. Phenom. Macrocycl. Chem..

[20] Xiao Z., Zhang Y., Niu Y., Ke Q., Kou X. (2021). Cyclodextrins as carriers for volatile aroma compounds: A review. Carbohydr. Polym..

[21] Kellici T.F., Chatziathanasiadou M.V., Diamantis D., Chatzikonstantinou A.V., Andreadelis I., Christodoulou E., Valsami G., Mavromoustakos T., Tzakos A.G. (2016). Mapping the interactions and bioactivity of quercetin–(2-hydroxypropyl)-*β*-cyclodextrin complex. Int. J. Pharm..

[22] Ntountaniotis D., Andreadelis I., Kellici T.F., Karageorgos V., Leonis G., Christodoulou E., Kiriakidi S., Becker-Baldus J., Stylos E.K., Chatziathanasiadou M.V. (2019). Host–Guest Interactions between Candesartan and Its Prodrug Candesartan Cilexetil in Complex with 2-Hydroxypropyl-*β*-cyclodextrin: On the Biological Potency for Angiotensin II Antagonism. Mol. Pharm..

[23] Liossi A.S., Ntountaniotis D., Kellici T.F., Chatziathanasiadou M.V., Megariotis G., Mania M., Becker-Baldus J., Kriechbaum M., Krajnc A., Christodoulou E. (2017). Exploring the interactions of irbesartan and irbesartan–2-hydroxypropyl-*β*-cyclodextrin complex with model membranes. Biochim. Biophys. Acta (BBA)-Biomembr..

[24] Hsu C.M., Yu S.C., Tsai F.J., Tsai Y. (2019). Characterization of in vitro and in vivo bioactivity of a ferulic acid-2-Hydroxypropyl-*β*-cyclodextrin inclusion complex. Colloids Surf. B Biointerfaces.

[25] Huang X., Guo H., Xie Q., Jin W., Zeng R., Hong Z., Zhang Y., Zhang Y. (2023). Preparation and Embedding Characterization of Hydroxypropyl-*β*-cyclodextrin/Menthyl Acetate Microcapsules with Enhanced Stability. Pharmaceutics.

[26] Rasheed A., Ashok K.C.K., Sravanthi V.V.N.S. (2008). Cyclodextrins as drug carrier molecule: A review. Sci. Pharm..

[27] Sliwinski E., Roubos P., Zoet F., Van Boekel M., Wouters J. (2003). Effects of heat on physicochemical properties of whey protein-stabilised emulsions. Colloids Surf. B Biointerfaces.

[28] Bae E., Lee S.J. (2008). Microencapsulation of avocado oil by spray drying using whey protein and maltodextrin. J. Microencapsul..

[29] Zhang S., Wang T. (2023). Preparation of enzymolysis porous corn starch composite microcapsules embedding organic sunscreen agents and its UV protection performance and stability. Carbohydr. Polym..

[30] Rodzik A., Pomastowski P., Sagandykova G.N., Buszewski B. (2020). Interactions of whey proteins with metal ions. Int. J. Mol. Sci..

[31] Hundre S.Y., Karthik P., Anandharamakrishnan C. (2015). Effect of whey protein isolate and *β*-cyclodextrin wall systems on stability of microencapsulated vanillin by spray–freeze drying method. Food Chem..

[32] Shalaby K.S., Ismail M.I., Lamprecht A. (2021). Cyclodextrin complex formation with water-soluble drugs: Conclusions from isothermal titration calorimetry and molecular modeling. AAPS PharmSciTech.

[33] Trott O., Olson A.J. (2010). AutoDock Vina: Improving the speed and accuracy of docking with a new scoring function, efficient optimization, and multithreading. J. Comput. Chem..

[34] Kim S.H., Zhang J., Jiang Y., Zhou H.M., Yan Y.B. (2006). Assisting the reactivation of guanidine hydrochloride-denatured aminoacylase by hydroxypropyl cyclodextrins. Biophys. J..

[35] Bastos M., Abian O., Johnson C.M., Ferreira-da Silva F., Vega S., Jimenez-Alesanco A., Ortega-Alarcon D., Velazquez-Campoy A. (2023). Isothermal titration calorimetry. Nat. Rev. Methods Primers.

[36] Bouchemal K., Mazzaferro S. (2012). How to conduct and interpret ITC experiments accurately for cyclodextrin–guest interactions. Drug Discov. Today.

[37] Bouchemal K., Couvreur P., Daoud-Mahammed S., Poupaert J., Gref R. (2009). A comprehensive study on the inclusion mechanism of benzophenone into supramolecular nanoassemblies prepared using two water-soluble associative polymers. J. Therm. Anal. Calorim..

[38] Daoud-Mahammed S., Couvreur P., Bouchemal K., Chéron M., Lebas G., Amiel C., Gref R. (2009). Cyclodextrin and polysaccharide-based nanogels: Entrapment of two hydrophobic molecules, benzophenone and tamoxifen. Biomacromolecules.

[39] Othman M., Bouchemal K., Couvreur P., Gref R. (2009). Microcalorimetric investigation on the formation of supramolecular nanoassemblies of associative polymers loaded with gadolinium chelate derivatives. Int. J. Pharm..

[40] Sajeesh S., Bouchemal K., Marsaud V., Vauthier C., Sharma C.P. (2010). Cyclodextrin complexed insulin encapsulated hydrogel microparticles: An oral delivery system for insulin. J. Control. Release.

[41] Mazzaferro S., Bouchemal K., Gallard J.F., Iorga B.I., Cheron M., Gueutin C., Steinmesse C., Ponchel G. (2011). Bivalent sequential binding of docetaxel to methyl-*β*-cyclodextrin. Int. J. Pharm..

[42] Ross P.D., Subramanian S. (1981). Thermodynamics of protein association reactions: Forces contributing to stability. Biochemistry.

[43] Yao H., Ke H., Zhang X., Pan S.J., Li M.S., Yang L.P., Schreckenbach G., Jiang W. (2018). Molecular recognition of hydrophilic molecules in water by combining the hydrophobic effect with hydrogen bonding. J. Am. Chem. Soc..

[44] Zhang Q., Chen Y., Geng F., Shen X. (2023). Characterization of Spray-Dried Microcapsules of Paprika Oleoresin Induced by Ultrasound and High-Pressure Homogenization: Physicochemical Properties and Storage Stability. Molecules.

[45] Saravanakumar K., Park S., Sathiyaseelan A., Kim K.N., Cho S.H., Mariadoss A.V.A., Wang M.H. (2021). Metabolite profiling of methanolic extract of Gardenia jaminoides by LC-MS/MS and GC-MS and its anti-diabetic, and anti-oxidant activities. Pharmaceuticals.

[46] Lu Y., Zhao R., Wang C., Zhang X., Wang C. (2022). Deciphering the non-covalent binding patterns of three whey proteins with rosmarinic acid by multi-spectroscopic, molecular docking and molecular dynamics simulation approaches. Food Hydrocoll..

[47] Zhang Y., Lu Y., Yang Y., Li S., Wang C., Wang C., Zhang T. (2021). Comparison of non-covalent binding interactions between three whey proteins and chlorogenic acid: Spectroscopic analysis and molecular docking. Food Biosci..

[48] Chatziathanasiadou M.V., Mavromoustakos T., Tzakos A.G. (2021). Unveiling the thermodynamic aspects of drug-cyclodextrin interactions through isothermal titration calorimetry. Supramolecules in Drug Discovery and Drug Delivery: Methods and Protocols.

[49] Prozeller D., Morsbach S., Landfester K. (2019). Isothermal titration calorimetry as a complementary method for investigating nanoparticle–protein interactions. Nanoscale.

